# The European gonococcal antimicrobial surveillance programme (Euro-GASP) appropriately reflects the antimicrobial resistance situation for *Neisseria gonorrhoeae* in the European Union/European Economic Area

**DOI:** 10.1186/s12879-019-4631-x

**Published:** 2019-12-10

**Authors:** Michelle J. Cole, Chantal Quinten, Susanne Jacobsson, Michaela Day, Andrew J. Amato-Gauci, Neil Woodford, Gianfranco Spiteri, Magnus Unemo, The Euro-GASP network, Angelika Stary, Maria Haller, Ruth Verbrugge, Tania Crucitti, Soteroulla Soteriou, Despo Pieridou, Susan Cowan, Steen Hoffmann, Jevgenia Epstein, Jelena Viktorova, Ndeindo Ndeikoundam, Agathe Goubard, Peter Kohl, Susanne Buder, Viviane Bremer, Eva Tzelepi, Vasileia Konte, Eszter Balla, Mária Dudás, Guðrún Sigmundsdóttir, Guðrún Svanborg Hauksdóttir, Derval Igoe, Brendan Crowley, Barbara Suligoi, Paola Stefanelli, Gatis Pakarna, Violeta Mavcutko, Christopher Barbara, Jackie Maistre Melillo, Alje Van Dam, Birgit Van Benthem, Ineke Linde, Hilde Kløvstad, Thea Bergheim, Slawomir Majewski, Beata Mlynarczyk-Bonikowska, Jacinta Azevedo, Maria José Borrego, Peter Pavlik, Peter Truska, Irena Klavs, Samo Jeverica, Julio Vazquez, Mercedes Diez, Inga Velicko, Magnus Unemo, Gwenda Hughes, Kirstine Eastick

**Affiliations:** 1 Antimicrobial Resistance and Healthcare Associated Infections (AMRHAI) Reference Unit, National Infection Service, Public Health England, London, UK; 2 European Centre for Disease Prevention and Control, Stockholm, Sweden; 3 WHO Collaborating Centre for Gonorrhoea and other STIs, Department of Laboratory Medicine, Faculty of Medicine and Health, Örebro University, Örebro, Sweden

**Keywords:** Gonorrhoea, Treatment, Antimicrobial resistance, Surveillance, European gonococcal antimicrobial surveillance programme (euro-GASP), Europe, European Union (EU), European Economic Area (EEA), Representativeness

## Abstract

**Background:**

European Gonococcal Antimicrobial Surveillance Programme (Euro-GASP) antimicrobial resistance (AMR) data are used to inform gonorrhoea treatment guidelines; therefore the data need to be robust and representative. We assessed the extent to which Euro-GASP reflects national measures of the AMR situation for *Neisseria gonorrhoeae* across the European Union/European Economic Area (EU/EEA).

**Methods:**

We compared data from Euro-GASP with published national gonococcal AMR data from 15 countries for azithromycin, cefixime and ciprofloxacin for the period 2009 to 2013 and performed Poisson regression to identify differences (*p* < 0.05) between the proportions of resistant isolates. The 2014 Euro-GASP AMR data for each country (*n* = 19) were weighted to account for differences in the distribution of patient characteristics between Euro-GASP and EU/EEA epidemiological gonorrhoea surveillance data. Data were compared to determine whether estimates of resistance levels differed with regards to the 5% threshold used to assess the clinical utility of first-line gonorrhoea treatments. We assessed the quality of decentralised testing by comparing AMR data for isolates tested both centrally and in the participating laboratories, and by evaluating external quality assessment (EQA) performance.

**Results:**

There was no significant difference for azithromycin, cefixime and ciprofloxacin resistance when Euro-GASP country data were compared with data from national reports. Weighting slightly altered the Euro-GASP AMR estimates (by between − 4.7 and 4.7% from the unweighted estimates). Weighting resulted in greater changes in estimates of resistance to azithromycin (from − 9.5 to 2.7%) and ciprofloxacin (from − 14.8 to 17.9%) in countries with low isolate numbers and low completeness of reporting (*n* = 3). Weighting caused AMR levels to fall below or above the 5% threshold for cefixime or azithromycin, respectively in only two countries. Susceptibility category data submitted from the decentralised Euro-GASP laboratories were concordant with the Euro-GASP data (> 90%). EQA performance was also good; < 5% of the minimum inhibitory concentration (MIC) results differed by > 4-fold from the modal MIC of the EQA isolate.

**Conclusions:**

The overall prevalence of AMR reported by Euro-GASP reflects closely the AMR situation for *N. gonorrhoeae* in the EU/EEA. Euro-GASP data can be used to provide robust AMR estimates to inform the European guideline for the management of gonorrhoea.

## Background

Empirical treatment of patients with confirmed or suspected gonorrhoea can reduce the duration of infection, prevent further onward transmission and alleviate the associated morbidity of gonorrhoea. As empirical therapy is administered in the absence of an antimicrobial susceptibility profile for the infecting isolate, the World Health Organization (WHO) recommends that the selected gonorrhoea treatment should have ≥95% probability of being effective i.e. 5% or fewer of gonococcal isolates are likely to be resistant to the antimicrobial used for first-line empirical treatment [1]. To ensure effective empirical therapy, surveillance of antimicrobial susceptibility in *Neisseria gonorrhoeae*, which informs revisions of gonorrhoea treatment guidelines, should be robust, reliable and quality-assured.

The antimicrobial susceptibility of the gonococcal population in the European Union/European Economic Area (EU/EEA) is monitored by the sentinel European Gonococcal Antimicrobial Surveillance Programme (Euro-GASP), which was initiated in 2004 [2] and has been funded, co-ordinated and expanded by the European Centre for Disease Prevention and Control (ECDC) since 2009. Euro-GASP data have twice informed changes to the first-line therapy recommended in the European guideline on the diagnosis and treatment of gonorrhoea; firstly by reporting the detection of high levels of ciprofloxacin resistance in the mid-2000s [3] and secondly by elucidating emerging resistance to cefixime in 2009/10 [4]. Originally, Euro-GASP used only centralised antimicrobial susceptibility testing, where 100–200 isolates from participating countries were sent to a central reference laboratory for testing [5]. However, since 2010, a more sustainable approach which includes decentralised testing, has been utilised [6]. Euro-GASP laboratories, usually one per country that also frequently perform their own national or regional (sub-national) gonococcal antimicrobial susceptibility surveillance, have been invited to participate in decentralised testing by submitting their own gonococcal antimicrobial susceptibility data. It is important that this shift from a centralised to decentralised testing strategy has a minimal impact on the longitudinal data. Quality criteria have therefore been agreed for countries to participate in decentralised testing using their own methods to test an agreed core antimicrobial panel: (i) a high concordance between the laboratories’ own antimicrobial susceptibility testing data and susceptibility data generated by Euro-GASP centralised susceptibility testing is required, and (ii) decentralised testing laboratories need to perform consistently well in the Euro-GASP external quality assessment (EQA) programme [7].

The provision of appropriate clinical, epidemiological and behavioural data for gonorrhoea patients linked to the antimicrobial susceptibilities of the collected gonococcal isolates is an essential component of Euro-GASP. Risk factor analysis can subsequently identify patient characteristics which are associated with infection with antimicrobial-resistant gonococcal isolates [8]. The ECDC additionally performs annual epidemiological surveillance for gonorrhoea infection [9], so patient data from the Euro-GASP can be compared with surveillance data enabling the representativeness of Euro-GASP to be investigated. Evaluation of the representativeness and validity of the antimicrobial susceptibility and epidemiologic data is essential to appropriately inform treatment guidelines.

The aim of this study was to evaluate the extent to which Euro-GASP data derived from a limited number of isolates per country and from both centralised and decentralised testing, reflect the antimicrobial resistance situation for *N. gonorrhoeae* as portrayed by data from routine national antimicrobial susceptibility surveillance systems and other directed antimicrobial susceptibility studies in the EU/EEA. Data from Euro-GASP were compared with data from other national antimicrobial susceptibility surveillance and survey sources in order to assess the validity of Euro-GASP data as a proxy for estimates based on larger national data sets. In addition, the representativeness of Euro-GASP data was assessed by applying weighting to Euro-GASP data to account for differences in the distribution of patient characteristics between the Euro-GASP patient data and the European epidemiological gonorrhoea surveillance data. In addition, we assessed the performance in the Euro-GASP EQA of countries moving from centralised to decentralised testing.

## Methods

### Comparison of Euro-GASP and national or regional gonococcal antimicrobial susceptibility data, 2009 to 2013

#### Data sources

During 2009 to 2013, 22 countries participated in Euro-GASP: Austria, Belgium, Cyprus (since 2010), Denmark, France, Germany, Greece, Hungary (since 2010), Iceland (since 2013), Ireland (since 2010), Italy, Latvia, Malta, the Netherlands, Norway, Portugal, Romania (2010–2011), Slovakia, Slovenia, Spain, Sweden and the United Kingdom. A PubMed search was performed to identify annual national or sub-national data from 2009 to 2013, produced from countries with laboratories participating in Euro-GASP. Keywords were (‘<country name>’) AND (‘gonorrhoea OR gonorrhoeae’). Additionally, a Google search using the identical keywords was performed. If no data were identified, an email was sent to the responsible Euro-GASP microbiologist in the specific country to enquire whether any published national or regional data were available.

These searches resulted in national datasets (see Additional file 1) derived from: (i) Euro-GASP laboratories which were also responsible for the production of national or sub-national gonococcal antimicrobial susceptibility data: Austria, Belgium, Denmark, Greece, Italy, Malta, Norway, Slovakia, Slovenia, Sweden and the United Kingdom (only England and Wales included in this paper), and (ii) Euro-GASP laboratories plus additional laboratories in the same country (The Netherlands, France) or solely non-Euro-GASP laboratories (Hungary and Germany). No published national or sub-national data were available for comparison from Cyprus, Iceland, Ireland, Latvia, Portugal, Romania and Spain.

Data on the countries participating in decentralised Euro-GASP, including methods for susceptibility testing, can be found in Additional file 1. In the centralised testing, Etest was performed for cefixime and ceftriaxone, and Etest or an agar dilution breakpoint method for azithromycin, ciprofloxacin, and spectinomycin. Etest or a full agar dilution series was performed for gentamicin, and β-lactamase production was identified using nitrocefin. The Euro-GASP results were reported to ECDC through The European Surveillance System (TESSy), along with associated pseudo-anonymised patient data (6).

In the present study, the total number of tested isolates, the number of resistant isolates and the percentage of resistant isolates were recorded for each year for azithromycin, cefixime, and ciprofloxacin from the Euro-GASP data and the national/sub-national data where available. Ceftriaxone (low numbers of resistant isolates), β-lactamase production (does not detect chromosomally-mediated penicillin resistance) and gentamicin (no resistance breakpoints defined) were not included.

For the analysis of weighted patient characteristics, epidemiological surveillance data were extracted from the TESSy database. These data are reported annually from EU/EEA Member States’ national surveillance systems. Only data from countries which also reported data in Euro-GASP were included in the dataset.

#### Statistical analysis

##### Comparison of Euro-GASP and national data, 2009 to 2013

The incidence rate defines the proportion of resistant isolates over the total number of isolates within a year. To assess statistically significant differences between the incidence rates from Euro-GASP and the national dataset, mixed-effect poisson regression analyses were used. For the poisson model, the dependent variable was the number of resistant isolates within 1 year, and the exposure command was the total number of isolates (incidence rate) within that year. The independent variable was the variable “system”, a binary variable for the source of the isolate data (Euro-GASP vs national). This allowed us to calculate whether the association between the two incidence rates was statistically significant. We included time as a random variable as the national and Euro-GASP datasets within 1 year are most likely more strongly correlated with each other than with other years.

To assess statistically significant differences at the EU/EEA level, the variable country was included as a random effect. A further analysis was performed at the EU/EEA level to determine whether the source of data at national level affected the primary analysis. The source of comparison data was defined as: (1) all data from the Euro-GASP laboratory and (2) Euro-GASP laboratory responsible for some or none of the Euro-GASP data. Country data with too few positive tests were excluded from all analyses (azithromycin and cefixime data from Hungary, and cefixime data from Germany).

Results were expressed as incidence rate ratios (IRR) with 95% confidence intervals (CI). The IRR provides a relative measure to assess the strength of association between the proportion of resistant isolates in the Euro-GASP versus national data. If the IRR is < 1.0, the national/regional data were composed of fewer resistant isolates than the Euro-GASP data. If the IRR is > 1.0, the national data were composed of more resistant isolates than the Euro-GASP data. If the IRR is 1.0, there was no difference between the national/regional data and the Euro-GASP data. No statistical significant differences between the two datasets (*p* > 0.05) suggests that the proportion of resistant isolates as reported through Euro-GASP reflect the national resistance levels.

##### Weighted patient characteristic, 2014

The Euro-GASP resistance data were weighted according to the distribution of specific patient characteristics as reported through the national epidemiological surveillance system and compared to the unweighted resistance levels to determine whether estimates of resistance levels differed with regards to the 5% threshold used to assess the clinical utility of first-line gonorrhoea treatments.

**Fig. 1:**
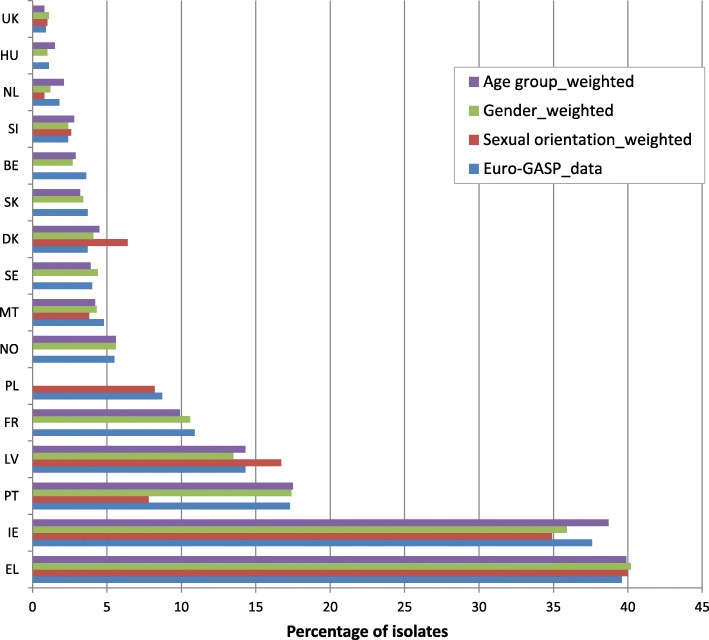
Percentage of azithromycin resistant isolates in Euro-GASP 2014 and estimates weighted for sexual orientation, gender and age group (< 25 years and ≥ 25 years)

**Fig. 2:**
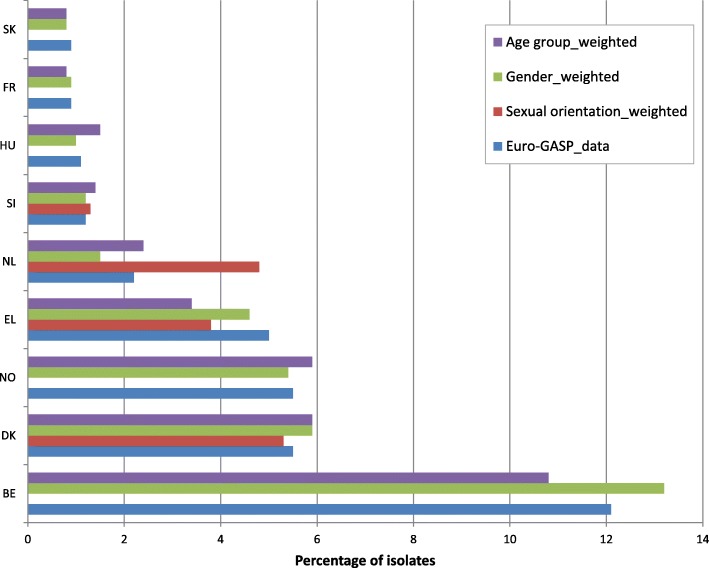
Percentage of cefixime resistant isolates in Euro-GASP 2014 and estimates weighted for sexual orientation, gender and age group (< 25 years and ≥ 25 years)

**Fig. 3:**
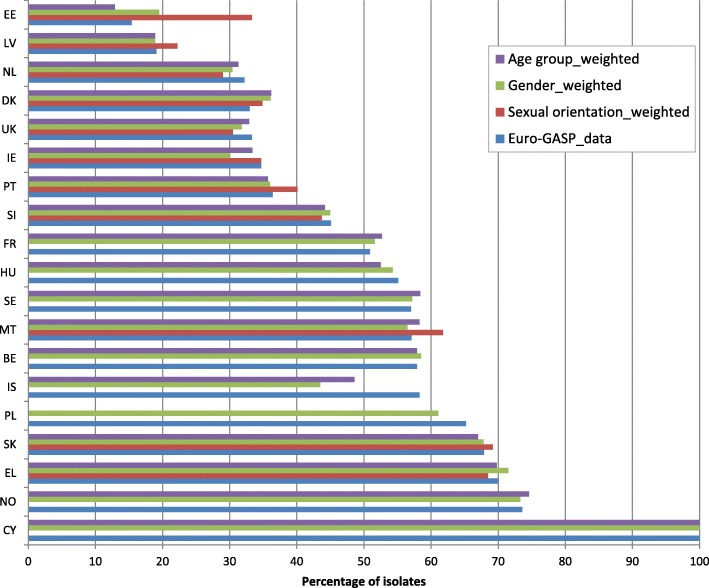
Percentage of ciprofloxacin resistant isolates in Euro-GASP 2014 and estimates weighted for sexual orientation, gender and age group (< 25 years and ≥ 25 years)

For 19 countries (Figs. 1, 2 and 3), weights were estimated, as previously described [10], to account for differences in age (≤25 years versus > 25 years), gender and sexual orientation (heterosexual versus men who have sex with men (MSM) between the Euro-GASP data and epidemiological surveillance data reported in 2014. All data were extracted from TESSy. For those countries which did not report the required patient data, no weighting was calculated. The weighted and unweighted resistance levels were then plotted for each country and data were compared to determine whether resistance levels shifted above or below the 5% threshold.

##### Quality assurance of decentralised antimicrobial susceptibility testing

In the first year of introduction of decentralised testing (2010), applications were received from the Euro-GASP laboratories in Belgium, Denmark, France, Italy, the Netherlands, Portugal, Spain, Sweden and the United Kingdom. The Euro-GASP laboratory in Greece joined decentralised testing in 2011. A further seven countries applied for decentralised testing until 2013: Austria, Cyprus, Iceland, Ireland, Malta, Norway and Slovenia. Parameters to further assure the quality of decentralised testing as well as data validity were investigated.

#### Comparison of antimicrobial susceptibility data

Available antimicrobial susceptibility data since 2006 from the laboratories’ own gonococcal antimicrobial susceptibility testing were matched per isolate to the centralised Euro-GASP data. In addition to β-lactamase, azithromycin, ciprofloxacin and ceftriaxone data which were available from 2006 in Euro-GASP, spectinomycin data were available from 2008, and cefixime and gentamicin data from 2009. Laboratories used either Etests or agar dilution to determine the minimum inhibitory concentration (MIC) of each antimicrobial and inferred susceptibility categories, i.e. susceptibility, intermediate susceptibility or resistance (see Additional file 1).

The percentage concordance of susceptibility categories was calculated for azithromycin, spectinomycin, and ciprofloxacin. If countries supplied MIC data with the susceptibility category data then the percentage concordance was calculated using EUCAST breakpoints [11] other than for ciprofloxacin pre-2013 (resistance MIC > 0.5 mg/L). For cefixime, ceftriaxone and gentamicin where full MICs were available, the concordance was calculated as the number of isolates within two doubling dilutions of the MICs. Positive and negative β-lactamase results were compared and a percentage concordance established. The acceptance rate for participation in decentralised testing, agreed by the Euro-GASP network, was that ≤5% of MIC results should differ by more than two MIC doubling dilutions and there should be ≥90% concordance for susceptibility categories and the β-lactamase results.

#### Comparison of EQA data

All available Euro-GASP EQA MIC data were assessed for each participating country up until the acceptance for performance of decentralised testing. Concordance was established by calculating the number of isolates that were within 4-fold (two doubling dilutions) of the modal MICs for the EQA isolates. To fulfil the decentralised quality criteria, laboratories were required to have ≤5% of MIC results that differed by more than 4-fold from the modal MIC of the EQA isolate.

## Results

### Comparison of Euro-GASP and national or sub-national data, 2009 to 2013

A total of 39,889 isolates (including both Euro-GASP and national/sub-national data) from 15 countries tested over the period 2009 to 2013 were compared for susceptibility to ciprofloxacin. This comparison of the pooled Euro-GASP data with the pooled national/sub-national antimicrobial susceptibility data for ciprofloxacin revealed a high level of concordance overall (*p* = 0.691) (Table 1), with only data from Germany (and only from 2011) provided by a non-Euro-GASP laboratory, displaying significantly more ciprofloxacin resistant isolates (by 50%) in the national/regional data compared with the Euro-GASP data (IRR 1.5, CI 1.06–1.97, *p* = 0.018) (Table 1). The comparison for azithromycin included 26,267 isolates from 10 countries. The concordance was also relatively high overall for azithromycin, with no significant differences between the two datasets for the majority of countries (7/10 countries) and overall between the national/sub-national data compared with the Euro-GASP data (*p* = 0.269) (Table 1). The country with the least amount of azithromycin resistant isolates compared to the Euro-GASP dataset was Austria with 86% less resistant isolates (IRR 0.14, CI 0.085–0.224, *p* < 0.0001) The Netherlands however, had more than double the number of resistant isolates compared to the Euro-GASP dataset (IRR 2.2, CI 1.32–3.76, *p* = 0.003) (Table 1). The cefixime analysis included 26,282 isolates from 10 countries. Concordance was lowest for cefixime susceptibility data, with no significant differences between the two datasets for half of the countries (5/10 countries). There was no significant difference between the national/regional data isolates and the overall Euro-GASP data for cefixime (*p* = 0.102) (Table 1). Adjusting for the source of comparison data in the model did not affect the findings for any of the antimicrobials.

**Table 1:** Poisson regression analysis assessing the relationship between Euro-GASP versus national antimicrobial susceptibility data to azithromycin, cefixime and ciprofloxaxin by country and overall, 2009 to 2013

	Azithromycin					Cefixime					Ciprofloxacin					Source ^b^
Country	IRR	*P* value	CI-lower	CI-upper	No. of tests/years	IRR	*P* value	CI-lower	CI-upper	No. of tests/years	IRR	*P* value	CI-lower	CI-upper	No. of tests/years	
Austria	0.138	**< 0.0001**	0.085	0.224	3456/4	0.052	**< 0.0001**	0.03	0.089	3454/4	1.000	0.995	0.885	1.129	3456/4	1
Belgium	0.68	0.079	0.442	1.046	3198/5	0.065	**< 0.0001**	0.18	0.241	1919/3	0.973	0.659	0.863	1.098	3198/5	1
Denmark	No national azithromycin susceptibility data available					No national cefixime susceptibility data available					1.006	0.923	0.895	1.131	2811/5	1
France	No national azithromycin susceptibility data available					0.871	0.747	0.375	2.019	5626/4	0.913	0.251	0.781	1.067	5628/4	2
Germany	6.085	0.083	0.791	46.79	321/1	Model not performed - too few positive isolates					1.447	**0.018**	1.064	1.968	321/1	2
Greece	1.078	0.781	0.634	1.832	425/3	1.282	0.415	0.705	2.331	505/3	1.008	0.917	0.87	1.167	1053/5	1
Hungary	Model not performed - too few positive isolates					Model not performed - too few positive isolates					0.98	0.839	0.803	1.194	776/3	2
Italy	0.944	0.794	0.615	1.451	1144/4	0.863	0.537	0.539	1.38	1170/4	1.003	0.972	0.859	1.171	1134/4	1
Malta	No resistant isolates					No national cefixime susceptibility data available					1.004	0.983	0.708	1.424	219/4	1
The Netherlands	2.23	**0.003**	1.324	3.757	4440/3	No national cefixime susceptibility data available					1.097	0.133	0.972	1.237	7695/5	2
Norway	1.389	0.152	0.886	2.177	594/2	2.22	**0.037**	1.051	4.687	594/2	1.063	0.596	0.848	1.334	594/2	1
Slovakia	No national azithromycin susceptibility data available					0.921	0.883	0.309	2.748	325/1	1.112	0.527	0.801	1.544	325/1	1
Slovenia	1.434	0.287	0.739	2.781	377/5	1.219	0.527	0.66	2.253	377/5	0.987	0.92	0.762	1.277	377/5	1
Sweden	1.818	**0.002**	1.252	2.638	4158/5	2.005	**0.006**	1.225	3.281	4158/5	0.956	0.459	0.849	1.077	4158/5	1
The United Kingdom	0.83	0.554	0.448	1.538	8154/5	1.706	**0.042**	1.02	2.852	8154/5	1.013	0.825	0.903	1.136	8154/5	1
All countries - univariate																
System^a^	1.09	0.269	0.935	1.271	26,267	0.863	0.101	0.724	1.029	26,282	1.008	0.697	0.969	1.049	39,889	
Source^b^	0.98	0.977	0.227	4.217	26,267	0.248	0.121	0.043	1.443	26,282	0.838	0.209	0.637	1.104	39,889	

^a^System = Euro-GASP data (1) vs. national data (2)

^b^Source = Source of comparison data; all data from Euro-GASP laboratory (1) vs. part or no data from Euro-GASP laboratory (2)

*IRR* incidence-rate ratio, *CI* 95% confidence interval. Significant values (< 0.05) in **bold**

### Weighted patient characteristics, 2014

For most countries (84.2%, 16/19) where comparative epidemiological surveillance data were available, weighting only slightly altered the estimates of overall antimicrobial resistance (from − 4.7 to 4.7% difference) (Figs. 1, 2 and 3). Larger differences were observed for azithromycin (from − 9.5 to 2.7%) (Fig. 1) and ciprofloxacin (from − 14.8 to 17.9%) (Fig. 3) for three countries with low isolate numbers and low completeness of reporting (Estonia and Iceland for ciprofloxacin and Portugal for azithromycin) [12, 13]. Critically, most (98.5%, 129/131) of the weighting did not cause estimated resistance levels to shift over or below the 5% threshold, which is used to assess the continued clinical utility of first-line antimicrobials for gonorrhoea empirical treatment. The exceptions were cefixime and azithromycin resistance in Greece and Denmark, respectively. For Greece, cefixime resistance changed from 5.0 to 3.8% for the sexual orientation-weighted estimates, to 4.6% for gender and to 3.4% for age group (Fig. 2). For Denmark, azithromycin resistance changed from 3.7 to 6.4% for the sexual orientation-weighted estimates (Fig. 1). Sexual orientation data was not available in either one of or both the Euro-GASP or the epidemiological surveillance datasets from Belgium, Cyprus, France, Hungary, Iceland, Norway, Poland and Sweden, and no age data were available from Poland in the epidemiological surveillance dataset.

### Comparison of decentralised and centralised susceptibility testing data

In total, 17 countries requested to participate in decentralised testing from 2010 to 2013 (Table 2).

**Table 2:** Percentage concordance of Euro-GASP centralised testing and laboratories’ own antimicrobial susceptibility testing results

Year	Ceftriaxone	Azithromycin	Cefixime	Ciprofloxacin	Spectinomycin	Gentamicin	β-lactamase
2006	87.6–100	95.3–100	–	49.5–99.1	–	–	96.3–100
2007	95.3–100	75.6–100	–	90.3–100	–	–	100
2008	93.8–100	41.7–100	–	94.2–100	100	–	98–100
2009	95.8–100	87–100	100	92.9–100	100	100	97.5–100
2010	93.8–100	89.1–100	97.1–100	92.6–100	100	100	98.2–100
2012	95.8–100	75^a^ – 100	95.8–100	80^b^ - 100	100	98.1–100	97.9–98.1
2013	95.4–100	89^a^ - 100	95.5–100	80^b^ - 100	100	97.3–100	99.1–100

The table contains data from; Austria (2012–2013, all antimicrobials); Belgium (2006–2009, no gentamicin or cefixime data); Cyprus (2012–2013, no spectinomycin, gentamicin or β-lactamase data); Denmark (2006–2009, ceftriaxone, ciprofloxacin and β-lactamase data only); France (2008–2010, no azithromycin or gentamicin data); Greece (2007–2010, no gentamicin data); Iceland (2012–2013, no spectinomycin, gentamicin or β-lactamase data); Ireland (2012, ceftriaxone, azithromycin and cefixime data only); Italy (2007, azithromycin and ciprofloxacin only, 2010–2011 all antimicrobials); Malta (2012–2013, no gentamicin or β-lactamase data); Norway (2012–2013, no β-lactamase data); Portugal (2009–2010, no azithromycin data); Slovenia (2011–2013, all antimicrobials); Spain (2006–2009, no cefixime or gentamicin data); Sweden (2006–2009, no cefixime or gentamicin data); The Netherlands (2006–2010, ceftriaxone, ciprofloxacin and β-lactamase data only) and the UK (2006–2010, no gentamicin data). Data from Slovenia was only available for 2011 so data is not shown, however all concordances for 2011 were within specified limits

^a^All discrepant isolates on the resistance breakpoint

^b^Only a very low number of isolates available for comparison from two labs, so 1–2 mismatches resulted in only 80% concordance, however overall concordance of the resistance category comparisons was ≥97%

#### Susceptibility category concordance

For all examined years, ≥90% concordance of the susceptibility categories was achieved for β-lactamase and spectinomycin testing (Table 2). For azithromycin, 28.6% (4/14) of countries testing for azithromycin achieved < 90% concordance between 2007 and 2010. However, 96.0% (48/50) of these discordant MIC results were within one doubling dilution of the resistance breakpoint, which can easily result in a different susceptibility category, and these were therefore accepted as comparable results. In 2012–2013, two laboratories achieved < 90% concordance for azithromycin, however, again all of the discordant MICs were on the resistance breakpoint, so the results were accepted. For ciprofloxacin, in 2006, one country had only 49.5% concordance of susceptibility category, and the laboratory could not explain this large discrepancy. However, this laboratory showed ciprofloxacin agreement > 90% for the four subsequent years, so participation was granted. Two countries achieved only 80% concordance for ciprofloxacin in either 2012 or 2013. However, only a very small number of isolates were available for comparison from the two laboratories, so only one to two mismatches resulted in the 80% concordance. The overall concordance of the susceptibility categories for all antibiotics tested was ≥97% and, accordingly, participation was granted.

#### MIC concordance

MIC concordance for cefixime and gentamicin was consistently > 95% (Table 2). Two laboratories had < 95% ceftriaxone MIC concordance for some of the years analysed. Thus, one laboratory achieved < 95% concordance for ceftriaxone MICs in 2006 and 2008 due to technical problems, which subsequently could be resolved by the laboratory. The differences were mainly (6/7) just one MIC doubling dilution more than the accepted two doubling dilutions. For this particular laboratory, the data from the subsequent years (2009 and 2010) showed excellent agreement (99.2 and 100% respectively) so participation was granted. One additional laboratory had < 95% ceftriaxone MIC concordance in 2010; however, this country reported on a very low number of isolates and only two out of 32 tested isolates were out of the accepted MIC range. As all other comparison data (100% agreement in 2010) and EQA data were acceptable, this laboratory was also accepted for decentralised testing.

#### Comparison of EQA data

All the Euro-GASP laboratories that requested to participate in decentralised testing had < 5% of all MICs measured for the EQA isolates that were greater than two MIC doubling dilutions from the modal MIC for the specific antimicrobial and EQA isolate (Table 3). The values ranged from 0 to 4.2% and overall 1.6% of measured MICs differed by more than two doubling dilutions from the modal MIC.

**Table 3:** Concordance in external quality assessment (EQA; MIC and β-lactamase testing results only)

Country	No. of susceptibility tests	% of isolates with MICs >2 doubling dilutions from the modal MIC (no.)
Austria	210	1.4 (3)
Belgium	500	0
Cyprus^a^	24	0
Denmark	420	0.5 (2)
France	460	2.6 (12)
Greece	291	0.3 (1)
Iceland^b^	40	0
Ireland^c^	30	0
Italy	430	4.2 (18)
Malta	230	1.3 (3)
Norway	180	0.6 (1)
Portugal	430	2.1 (9)
Slovenia	730	0.6 (4)
Spain	520	2.5 (13)
Sweden	490	1.4 (7)
The Netherlands	210	0
United Kingdom	510	3.1 (16)

^a^Low number of isolates available and previously used disc diffusion method

^b^Recent Euro-GASP member and very low number of isolates available

^c^Previously used disc diffusion method for EQA and therefore low number of EQA MIC results available

## Discussion

The overall antimicrobial susceptibility data from the Euro-GASP laboratories did not significantly differ from the national/sub-national susceptibility data. The azithromycin and cefixime differences in a small number of countries were possibly due to isolates with MICs close to or on the resistance breakpoint, which result in greater variability in testing and interpretation of the susceptibility categories. In addition the activity of azithromycin is sensitive to pH changes in the agar medium, which can affect MICs and subsequent clinical interpretation [14]. Some differences between the datasets are expected, particularly because Euro-GASP overall includes a smaller number of isolates than the national surveillance, the criteria for selection of isolates differ, and different susceptibility testing methodologies can be used. In addition, the national antimicrobial data are generated from several laboratories in some countries meaning that the data may be less quality-assured compared to the Euro-GASP requirements. On the other hand, national data from a larger number of laboratories are likely to be more geographically representative. A limitation of this analysis is the variation in the number of years for which data were available for comparison: from one to 5 years depending upon the country. In addition, the number of tested isolates varied by country and year and smaller numbers meant wide confidence intervals for some antimicrobials. Increased availability of data at the national level would allow for more confidence in analysis and interpretation of these results. In addition, it is possible that some isolates where included both in the Euro-GASP and national/regional datasets, which would lead to dependence between the two datasets. This was not accounted for in the analysis since data were available aggregated to the system/lab level for the national/sub-national dataset.

There were minor differences between the Euro-GASP antimicrobial resistance data and the weighted resistance data in respect to the available patient characteristic data from the epidemiological surveillance [9]. In general, weighting of the Euro-GASP data did not provide significantly different estimates of resistance levels that would impact on the 5% threshold used to assess the continued clinical utility of first-line antimicrobials in the empirical treatment of gonorrhoea. However, one limitation of this analysis is the variations in reporting, such as sentinel epidemiological surveillance for gonorrhoea in some countries [12], which means the data used for weighting are not necessarily the true population of gonorrhoea patients or the same population Euro-GASP isolates were obtained from. Another limitation was the lack of some patient data in the epidemiological surveillance and/or the Euro-GASP dataset. This was particularly a problem for the epidemiological variable sexual orientation, which was not reported by eight countries and this also means that combined weighting using all three variables (age, gender and sexual orientation) was not possible due to the low data completeness. Consequently, ensuring and comprehensively assessing a truly representative sample will not be possible until country reporting is improved, which is an ongoing activity. Furthermore, the heterogeneity of the epidemiological surveillance, as previously discussed [8], is a weakness in this analysis, and in the absence of complete comprehensive epidemiological surveillance in all countries, weighting of the Euro-GASP data might not be of any significant value, even with a complete Euro-GASP dataset.

Only a few previous studies have investigated the representativeness of national or international GASPs. Comparisons of the distribution of patient epidemiological data in both the Euro-GASP and the ECDC epidemiological surveillance data were investigated previously [8] and showed that the distribution of the reported patient characteristics of age and sexual orientation between the two surveillance systems significantly differed, except for the proportion of MSM, and that male heterosexuals were over-represented in the Euro-GASP data set. However the weighted analysis in the current study investigated data from individual countries which submitted both patient data linked to the gonococcal isolates, and to the epidemiological surveillance, and these showed high concordance. A study from the Gonococcal Resistance to Antimicrobials Surveillance Programme (GRASP) in England and Wales also used weighting to estimate resistance prevalence [10]. Their investigations revealed that the GRASP sample, which is also based on annual 3-monthly sentinel surveillance, provides reliable estimates of resistance levels and weighted estimates would not have changed national recommendations to change treatment guidelines. Accordingly, the findings of the present study evaluating Euro-GASP are in full concordance with the findings of the national GRASP study. Another British study [15] investigated two sampling methods over 3 months; every 5th isolate compared with the first 20 each month. Analysis of the susceptibility profiles of the gonococcal isolates revealed no significant difference between the two sampling methods. The authors concluded that major changes can be monitored in the gonococcal population using a small sample of the population. This is in concordance with our results where the Euro-GASP data, with an average of 100 isolates per country/year, and the national data frequently compiled using much larger samples were mainly highly concordant. Two studies have evaluated the number of isolates tested in a gonococcal antimicrobial susceptibility survey compared to the number of reported gonorrhoea cases and both have shown that the use of nucleic acid amplifications tests (NAATs) for detection of *N. gonorrhoeae*, as opposed to culture, had an impact on representativeness [16, 17]. In Euro-GASP 2015, the overall number of isolates tested was 3% of the total number of cases reported via the epidemiological surveillance for the same countries [18]. The main reasons for this are the lack of widely available culture possibilities in several countries and the broad implementation of gonococcal NAATs. For comparison, in the US Gonococcal Isolate Surveillance Project (GISP), isolates from approximately 1.5% of the reported gonorrhoea cases are tested for antimicrobial susceptibility and only isolates from urethral samples from males are included [19]. In Euro-GASP, isolates are collected from both males and females, and from all anatomical sites of infection. For some countries with low numbers of gonorrhoea cases, the numbers of isolates submitted to Euro-GASP are similar to the overall number of reported gonorrhoea cases, whereas the numbers of isolates are capped for the other Euro-GASP countries for practical and resource reasons.

The advantages of decentralised testing within Euro-GASP include decreased cost, enhanced sustainability, improved timeliness, increased efficiency, and encouragement of more laboratories to test more isolates in a quality-assured manner and focus on most appropriate antimicrobials used in treatment. The antimicrobial susceptibility data submitted from the decentralised Euro-GASP laboratories were also overall highly concordant with the data generated by the Euro-GASP centralised testing. This, along with appropriate EQA data, supports the continuation of Euro-GASP laboratories’ participating in decentralised testing, even when slightly different methodologies are used. Likely reasons for the few identified discrepancies include differences in agar media, size of inoculum, incubation (atmosphere, temperature and time), as well as the subjective reading and interpretation of the antimicrobial susceptibility testing. To ensure continual confidence in the quality of the decentralised data, all decentralised laboratories are requested to use the same quality control strain panel [18] and continue to participate in the EQA [20]. Any problems identified in the EQA are promptly investigated further. Potential problems with decentralised testing include less control over the surveillance process, e.g. delays in submitting data from countries that delays publication of the results, and that laboratories may change to different methods or stop their own susceptibility testing particularly if resources locally are reduced. Work is ongoing to switch those countries that currently rely on centralised testing to the decentralised model through training and country visits.

The added value of Euro-GASP, as opposed to relying upon just the national reports, includes the generation of regional data and standardised reporting via TESSy, including a drive for improved patient data reporting so appropriate risk analysis can be performed. The overall quality of the network is enhanced by methodological standardisation, participation in the EQA and delivery of training. Euro-GASP is performed annually as opposed to, at times, episodic analysis from some countries. Not all countries produce annual reports but rely upon publications which describe the antimicrobial resistance (AMR) situation over a number of combined years, and many have limited epidemiological analysis. Euro-GASP is a collaborative network that can share ideas and expertise, and allows a platform to highlight emerging issues as well as perform additional projects and surveys [21–23].

As antimicrobial resistance in *N. gonorrhoeae* is now a global problem, a reliable global picture is required. International gonococcal antimicrobial susceptibility surveillance is a goal of the WHO and is co-ordinated by the WHO Global GASP [24]. Dillon [25] has described the difficulties faced with international surveillance, such as representativeness, timely reporting of data, ability to publish data, lack of available cultures, funding, and substantial differences between regional surveillance programmes. When comparing international antimicrobial susceptibility data, it is important that the same breakpoints are used, and even though this is no longer an issue for the EU/EEA where the breakpoints have been harmonised [11], variation in interpretive criteria still confounds comparisons with other regions. An internationally agreed methodology and harmonised breakpoints would be very useful to compare gonococcal AMR data from around the world, as would the use of identical WHO reference strains [26]. The main challenge for any GASP is that NAATs are rapidly replacing culture for detection of *N. gonorrhoeae* internationally. Although antimicrobial resistance determinants detected by PCRs or sequencing will most likely be informing antimicrobial resistance surveillance programmes more frequently in the future, molecular prediction of antimicrobial resistance in *N. gonorrhoeae* currently has important inherent limitations, including that new or unknown resistance determinants are not detected, and the suboptimal correlates between molecular resistance determinants and MICs of several antimicrobials [27]. Accordingly, phenotypic antimicrobial susceptibility surveillance, in the future supplemented with molecular resistance prediction, will remain the basis for informing revisions of treatment guidelines and it is very important to maintain and strengthen capacity to perform gonococcal culture and antimicrobial susceptibility testing globally.

## Conclusions

The key functions of Euro-GASP are to detect emerging and developing antimicrobial resistance, establish trends of antimicrobial resistance over time, and inform refinements of treatment guidelines. This study shows that the prevalence of antimicrobial resistance reported by the Euro-GASP sentinel surveillance system appropriately reflects the antimicrobial resistance situation in the EU/EEA, and Euro-GASP can therefore provide robust resistance estimates to inform the European guideline for the diagnosis and treatment of gonorrhoea [4].

## Supplementary information

**Additional file 1.** Data source of national/sub-national antimicrobial susceptibility data and methodology used to establish decentralised testing and/or national/sub-national data.

## Data Availability

The data that support the findings of this study are available from the European Centre for Disease Prevention and Control, but restrictions apply to the availability of these data, which were used under license for the current study, and so are not publicly available. Data may however be available from the authors upon reasonable request and with permission of the European Centre for Disease Prevention and Control.

## Abbreviations

AMR: Antimicrobial resistance
CI: Confidence interval
ECDC: European Centre for Disease Prevention and Control
EEA: European Economic Area
EQA: External quality assessment
EU: European Union
Euro-GASP: European Gonococcal Antimicrobial Surveillance Programme
GASP: Gonococcal Antimicrobials Surveillance Programme
GISP: Gonococcal Isolate Surveillance Project
GRASP: Gonococcal Resistance to Antimicrobials Surveillance Programme
IRR: Incidence rate ratio
MIC: Minimum inhibitory concentration
MSM: Men who have sex with men
NAATs: nucleic acid amplifications tests
No.: Number
TESSy: The European Surveillance System
UK: United Kingdom
US: United States
WHO: World Health Organization
