# Supplementary material for: The European gonococcal antimicrobial surveillance programme (Euro-GASP) appropriately reflects the antimicrobial resistance situation for Neisseria gonorrhoeae in the European Union/European Economic Area
Source: BMC Infect Dis. 2019 Dec 10;19:1040. doi: 10.1186/s12879-019-4631-x (PMC6902330; doi:10.1186/s12879-019-4631-x)
Supplement: Supplementary file 1 — Additional file 1. Data source of national/sub-national antimicrobial susceptibility data and methodology used to establish decentralised testing and/or national/sub-national data. [file 12879_2019_4631_MOESM1_ESM.docx]

**Additional file 1: Data source of national/sub-national antimicrobial susceptibility data and methodology used to establish decentralised testing and/or national/sub-national data**

|  | **National/sub-national susceptibility data** | | | **Decentralised Euro-GASP/National susceptibility testing methods (2013)** | | | | | |
| --- | --- | --- | --- | --- | --- | --- | --- | --- | --- |
| **Country** | **Data source of data** | **Laboratory** | **Years available** | **Testing model** | **Method** | **Agar** | **Breakpoints** | | **Year decentralised** |
| Austria | <http://onlinelibrary.wiley.com/doi/10.1111/ddg.12816/abstract;jsessionid=43700C73BD1B2E1DC7E0212D4E06AD66.f03t03> | 1 | 2010-2013 | Decentralised | Agar dilution/Etest | GC | | EUCAST | 2014 |
| Belgium | 2009 – 2011 <https://www.wiv-isp.be/epidemio/epinl/plabnl/plabannl/tt_036n.htm>  2012-13 reports directly from Euro-GASP collaborator | 1 | 2009-2013 | Decentralised | Agar dilution | GC | | CLSI | 2010 |
| Cyprus | None available | | | Decentralised | Etest | GC | | EUCAST | 2013 |
| Denmark | 2009: <https://www.ssi.dk/~/media/Indhold/EN%20-%20engelsk/EPI-NEWS/2009/pdf/EPI-NEWS%20-%202009%20-%20No%2022.ashx>  2010: <https://www.ssi.dk/English/News/EPI-NEWS/2011/No%2034a%20-%202011.aspx>  2011: <https://www.ssi.dk/English/News/EPI-NEWS/2012/No%2036%20-%202012.aspx>  2012: <https://www.ssi.dk/English/News/EPI-NEWS/2013/No%2035%20-%202013.aspx>  2013: <https://www.ssi.dk/English/News/EPI-NEWS/2014/No%2035%20-%202014.aspx> | 1 | 2009-2013 | Decentralised | Etest | Chocolate | | EUCAST | 2010 |
| France | <http://www.eurosurveillance.org/content/10.2807/1560-7917.ES2014.19.34.20885> | 2 | 2009-2012 | Decentralised | Etest | Chocolate | | EUCAST | 2010 |
| Germany | <http://www.sciencedirect.com/science/article/pii/S143842211400037X?via%3Dihub> | 2 | 2011 | Centralised | Etest | Muller Hinton chocolate | | EUCAST |  |
| Greece | National reports directly from Euro-GASP collaborator | 1 | 2009-2013 | Decentralised | Etest | GC | | EUCAST | 2011 |
| Hungary | <https://bmcinfectdis.biomedcentral.com/articles/10.1186/1471-2334-14-433> | 2 | 2010-2012 | Centralised | Liofilchem strips | chocolate | | EUCAST |  |
| Iceland | None available | | | Decentralised | Etest | GC | | EUCAST | 2013 |
| Ireland | None available | | | Decentralised | Etest | GC | | EUCAST | 2013 |
| Italy | <http://aac.asm.org/content/58/10/5871.long> | 1 | 2009-2012 | Decentralised | Agar dilution/Etest | GC/Thayer-martin | | EUCAST | 2010 |
| Latvia | None available | | | Centralised |  |  | |  |  |
| Malta | WHONET data directly from Euro-GASP collaborator | 1 | 2010-2013 | Decentralised | Etest | GC | | EUCAST | 2014 |
| The Netherlands | <http://www.rivm.nl/Documenten_en_publicaties/Algemeen_Actueel/Uitgaven/Infectieziekten/Voortgangsrapportages_GRAS/Download/Voortgangsrapportage_GRAS_november_2014.org>  2012:<http://www.rivm.nl/dsresource?objectid=4b6aa1d9-3c11-46d0-aa95-762d3c1bdded&type=org&disposition=inline>  2013: <http://www.rivm.nl/dsresource?objectid=a88fd4f6-9bd9-4599-8463-49fde9d03b5e&type=org&disposition=inline> | 2 | 2009-2013 | Decentralised | Etest | GC | | EUCAST | 2010 |
| Norway | 2013: <http://wwweng.vetinst.no/eng/content/download/14065/170822/file/NORM%20NORM-VET%202013.pdf>  2010: <http://wwweng.vetinst.no/eng/content/download/8429/101343/file/NORM_VET_2010.pdf> | 1 | 2010 and 2013 | Decentralised | Etest | GC | | EUCAST | 2014 |
| Portugal | None available | | | Decentralised | Etest | GC | | EUCAST | 2010 |
| Romania | None available | | | Centralised |  |  | |  |  |
| Slovenia | <https://academic.oup.com/jac/article-lookup/doi/10.1093/jac/dku026> | 1 | 2009-2013 | Decentralised – Etest | Etest | GC | | EUCAST | 2014 |
| Slovakia | National reports directly from Euro-GASP collaborator | 1 | 2013 | Centralised | Etest | DST | |  |  |
| Spain | None available | | | Decentralised | Agar dilution |  | |  | 2010 |
| Sweden | <http://www.sva.se/globalassets/redesign2011/pdf/om_sva/publikationer/swedres_svarm2013.pdf> | 1 | 2009-2013 | Decentralised – Etest | Etest | GC | | EUCAST | 2010 |
| The United Kingdom | <https://www.gov.uk/government/uploads/system/uploads/attachment_data/file/368477/GRASP_Report_2013.pdf> * | 1 | 2009-2013 | Decentralised – MIC | Agar dilution | DST | | GRASP | 2010 |

Laboratory; Euro-GASP laboratory=1; Non-Euro-GASP laboratory=2

* Additional data from personal communication as absolute numbers not available from report.
